# Tubular secretion in chronic kidney disease: from established physiology to clinical utility

**DOI:** 10.3389/fcell.2026.1945081

**Published:** 2026-09-10

**Authors:** Natalia M. Stepanova

**Affiliations:** Medical Center LLC “Nephrocenter”, Kyiv, Ukraine

**Keywords:** biomarkers, chronic kidney disease, drug transporters, proximal tubule, organic anion transporters, precision medicine, tubular secretion, uremic toxins

## Abstract

In recent years, growing clinical evidence has renewed interest in tubular secretion as a distinct and underappreciated domain of kidney function in chronic kidney disease (CKD). Secretory clearance may provide information beyond estimated glomerular filtration rate and albuminuria, especially for kidney function decline, CKD progression, mortality, and selected adverse events. However, the evidence remains fragmented. Available studies differ in solute selection, sampling methods, analytical platforms, and outcome definitions. It also remains unclear how secretion measures should be translated into clinical care. This review integrates physiological, analytical, prognostic, and implementation evidence to define the requirements for developing clinically interpretable secretory biomarkers. It proposes a translational pathway from assay standardization and secretory phenotype validation to prospective testing of secretion-guided risk assessment and medication management.

## Introduction

Chronic kidney disease (CKD) is staged mainly by estimated glomerular filtration rate (eGFR) and albuminuria, but these measures do not capture all kidney functions that may influence solute retention, medication exposure, and outcomes (KDIGO CKD Work Group, 2024). This framework is practical, standardized, and prognostic. However, it is also filtration-centered. Patients with similar eGFR and albuminuria can differ substantially in uremic solute burden, drug tolerance, adverse-event risk, and rate of kidney function decline (Suchy-Dicey et al., 2016; Wang and Kestenbaum, 2018; McDonnell et al., 2025). This heterogeneity suggests that clinically relevant non-glomerular functions remain undermeasured in routine CKD care.

Tubular secretion is one such function. It is the active proximal tubular transport of selected endogenous metabolites, protein-bound uremic solutes, gut-derived compounds, xenobiotics, and medications from blood into urine (Wang and Kestenbaum, 2018; Bush et al., 2020; Granda et al., 2023). Because many secreted solutes are protein-bound or transporter-dependent, their elimination depends substantially on proximal tubular transport and may not be adequately reflected by filtration markers (Wu et al., 2017; Wang and Kestenbaum, 2018; Bush et al., 2020). Kidney clearance of an endogenous secretory solute, however, is not a direct measure of pure tubular secretion. It reflects net renal handling and may include glomerular filtration and, for some compounds, tubular reabsorption (Thompson and Joy, 2022). Plasma concentration represents another, distinct measure. It reflects circulating solute burden and is influenced by renal clearance as well as solute production and other nonrenal determinants (Wang and Kestenbaum, 2018; Zaidan and Nazzal, 2022). Thus, renal clearance, tubular secretion, and circulating solute burden should not be considered interchangeable. In this review, “tubular secretion” refers to the active transport process, whereas “secretory clearance” refers to the kidney clearance of solutes known to undergo substantial tubular secretion.

Tubular secretory function may be particularly vulnerable in CKD. Tubular injury, interstitial fibrosis, inflammation, hypoxia, altered transporter activity, and polypharmacy may impair secretory capacity (Suchy-Dicey et al., 2016; Wang and Kestenbaum, 2018; Tan et al., 2022). In advanced CKD, clearances of several actively secreted organic solutes may be reduced disproportionately, which may promote the accumulation of uremic compounds and alter exposure to transporter-dependent medications (Mair et al., 2021). These observations have renewed interest in tubular secretion as a potential marker of kidney function and as a clinically relevant pathway for medication safety.

Recent analytical advances have made tubular secretory function increasingly assessable in human studies. Targeted mass spectrometry and metabolomic approaches now allow simultaneous assessment of multiple endogenous secretory solutes in plasma and urine (Suchy-Dicey et al., 2016; Wang and Kestenbaum, 2018). Cohort data now link lower secretory clearance with kidney function decline, CKD progression, mortality, adverse events, and selected cardiovascular outcomes (Chen et al., 2020; 2021b; Ascher et al., 2022; Bullen et al., 2022; Granda et al., 2022). However, these data remain insufficient to support routine secretory testing for CKD staging or medication dosing. Available studies differ in solute selection, sampling methods, analytical platforms, and outcome definitions. Plasma concentrations, spot urine-to-plasma ratios, timed clearances, and drug-probe approaches also measure related but noninterchangeable aspects of secretion (Suchy-Dicey et al., 2016; Rivara et al., 2017; Chen et al., 2021a; Ascher et al., 2022; Bullen et al., 2022).

Previous reviews have addressed the physiological significance and potential applications of proximal tubular secretory clearance, broader biomarkers of tubular health, and renal drug transporters (Yin and Wang, 2016; Wang and Kestenbaum, 2018; Thompson and Joy, 2022; Spicher et al., 2025). However, none has integrated contemporary measurement approaches, human prognostic evidence, and the requirements for establishing clinical utility of secretory biomarkers in CKD. Therefore, the present review integrates these areas within a clinical-translation framework that distinguishes measurable secretion, clinical validity, and clinical utility. It examines which candidate biomarkers are most informative, why current secretion measures are not interchangeable, and what analytical, prognostic, and prospective evidence is required before secretory profiling can inform CKD risk assessment, medication safety, or precision nephrology.

## Proximal tubular secretion as a distinct kidney function

Glomerular filtration and tubular secretion both remove solutes, but they are different kidney functions. eGFR primarily reflects the filtration of small or freely circulating solutes across the glomerular filtration barrier (Pottel et al., 2024). By contrast, tubular secretion is an active, vectorial, and transporter-mediated process that moves selected solutes from peritubular blood into the tubular lumen. It requires basolateral uptake across the peritubular membrane, intracellular handling, and apical efflux into urine. Secretory clearance therefore depends on epithelial polarity, transporter expression and activity, peritubular solute delivery, substrate competition, and cellular energy metabolism (Pottel et al., 2024; Alamilla-Sanchez et al., 2025).

This distinction is significant in CKD, as filtration and secretion may not decline simultaneously. Filtration can decrease due to nephron loss or changes in glomerular hemodynamics (Nankivell et al., 2020; Kolesnyk and Stepanova, 2024; Pottel et al., 2024). Proximal tubular injury, interstitial fibrosis, inflammation, hypoxia, mitochondrial dysfunction, altered transporter regulation, or transporter inhibition may lower secretory capacity (Suchy-Dicey et al., 2016; Wang and Kestenbaum, 2018; Alamilla-Sanchez et al., 2025). These mechanisms may overlap with those that reduce GFR, but they are not identical. As a result, residual secretory function may not be proportional to eGFR. This provides a biological basis for why patients with similar filtration markers may differ in uremic solute burden, medication tolerance, and adverse-event risk.

**FIGURE 1:**
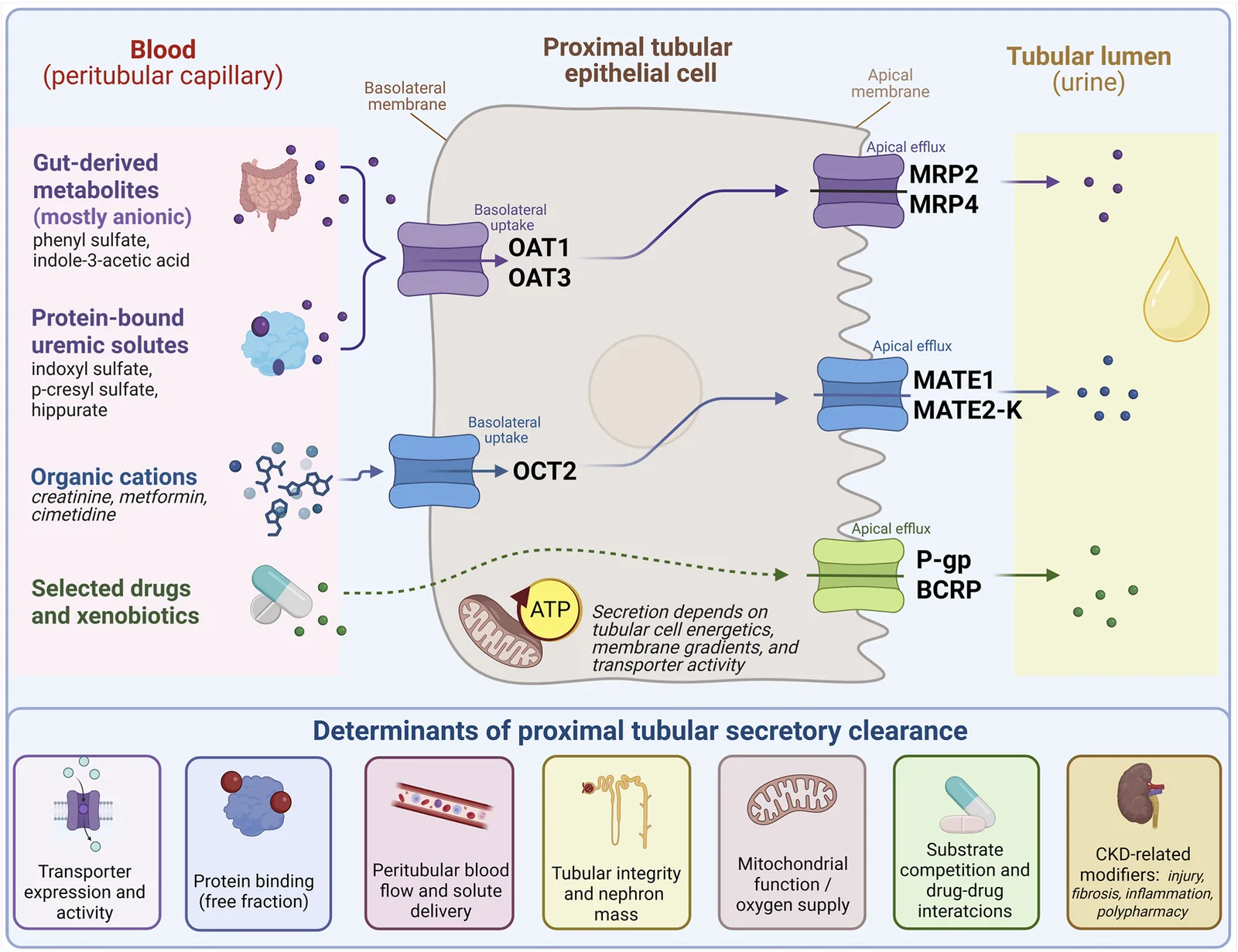
Proximal tubular secretion: representative transport pathways and determinants of clearance. Proximal tubular secretion moves selected solutes and drugs from peritubular blood into the tubular lumen. Organic anions, including many protein-bound and gut-derived uremic solutes, are taken up mainly by OAT1/OAT3 and exported by apical efflux transporters such as MRP2/MRP4. Organic cations are taken up by OCT2 and excreted mainly through MATE1/MATE2-K. Other apical transporters, including P-gp and BCRP, help handle selected drugs and xenobiotics. Secretory clearance is influenced by transporter activity, protein binding, tubular integrity, peritubular solute delivery, mitochondrial function, substrate competition, drug-drug interactions, and CKD-related tubular injury. Abbreviations: BCRP, breast cancer resistance protein; CKD, chronic kidney disease; MATE, multidrug and toxin extrusion protein; MRP, multidrug resistance-associated protein; OAT, organic anion transporter; OCT2, organic cation transporter 2; P-gp, P-glycoprotein. Created in BioRender. Stepanova, N. (2026) https://BioRender.com/dndhnfi License: BioRender Academic Publication License, Agreement No. YB2A5UJOOK Source: BioRender.

The proximal tubule is the main site of renal secretion. Its basolateral membrane faces the peritubular capillary. Its apical membrane faces the tubular lumen (Wang and Kestenbaum, 2018; Alamilla-Sanchez et al., 2025). Secreted solutes move across these two membranes through coordinated uptake and efflux pathways (Suchy-Dicey et al., 2016; Wang and Kestenbaum, 2018). Figure 1 shows the main transporter pathways, substrate classes, and determinants of secretory clearance.

Several transporter systems support proximal tubular secretion. Organic anion transporters 1 and 3 (OAT1/OAT3), organic cation transporter 2 (OCT2), multidrug and toxin extrusion proteins 1 and 2-K (MATE1/MATE2-K), multidrug resistance-associated proteins 2 and 4 (MRP2/MRP4), breast cancer resistance protein (BCRP), and P-glycoprotein (P-gp) contribute to basolateral uptake and apical efflux (Suchy-Dicey et al., 2016; Wu et al., 2017; Alamilla-Sanchez et al., 2025). Together, these systems move selected compounds from blood to urine, including drugs and solutes that are poorly cleared by filtration alone. These pathways handle overlapping but nonidentical groups of endogenous metabolites, uremic solutes, xenobiotics, and medications. Table 1 summarizes the main proximal tubular transport pathways relevant to CKD.

**TABLE 1:** Main proximal tubular secretory pathways relevant to CKD.

Pathway	Main role	Nephron location	Representative substrates	CKD relevance and limitation
OAT1/OAT3 (Yin and Wang, 2016; Wu et al., 2017; Bush et al., 2020)	Basolateral uptake of organic anions from peritubular blood	Proximal tubule, basolateral membrane	Hippurate, indoxyl sulfate, p-cresyl sulfate, beta-lactam antibiotics, antiviral drugs, loop and thiazide diuretics	Central pathway for anionic secretion. Interpretation is limited because substrates overlap and solute levels may reflect diet, microbiome activity, protein binding, and nonrenal metabolism
OCT2–MATE1/MATE2-K (Yin and Wang, 2016)	Basolateral uptake and apical efflux of organic cations	Proximal tubule; OCT2 basolateral, MATE1/MATE2-K apical	Creatinine, metformin, cimetidine, and other cationic drugs	Relevant to cationic drug handling and transporter-mediated creatinine secretion. Hard to assess from endogenous markers alone
MRP2/MRP4 (Masereeuw and Russel, 2012)	Apical efflux of organic anions, conjugates, and selected drugs	Proximal tubule, apical membrane	Sulfate and glucuronide conjugates, antiviral drugs, and selected anionic solutes	Supports urinary efflux after basolateral uptake. Apical efflux is rarely measured directly in human CKD studies
BCRP and P-gp (Masereeuw and Russel, 2012)	Apical efflux of selected drugs and xenobiotics	Proximal tubule, apical membrane	Urate, digoxin, selected anticancer drugs, immunosuppressants, and antiviral drugs	Relevant to drug exposure and drug-drug interactions. Kidney-specific interpretation is difficult because these transporters are also expressed outside the kidney

Abbreviations: BCRP, breast cancer resistance protein; CKD, chronic kidney disease; MATE, multidrug and toxin extrusion protein; MRP, multidrug resistance-associated protein; OAT, organic anion transporter; OCT2, organic cation transporter 2; P-gp, P-glycoprotein.

Secretion also requires a cellular energy supply, though most proximal tubular transporters are not ATP pumps. They function based on ion gradients, membrane potential, intracellular exchange systems, and ATP-dependent cellular homeostasis (Suchy-Dicey et al., 2016; Wang and Kestenbaum, 2018; Łapczuk-Romańska et al., 2023). In proximal tubular cells, which have high mitochondrial activity and require ample oxygen, hypoxia or metabolic stress may impair transport capacity even when filtration is relatively preserved (Wei et al., 2019; Li et al., 2021). This metabolic dependence provides another mechanism by which secretory function may diverge from eGFR in CKD.

Tubular secretion handles a heterogeneous group of endogenous and exogenous compounds. These include organic acids, organic bases, conjugated metabolites, gut-derived solutes, environmental xenobiotics, and many medications (Suchy-Dicey et al., 2016; Wang and Kestenbaum, 2018). Many of these compounds arise from host metabolism, gut microbial metabolism, or both. Thus, the proximal tubule acts as an interface between the circulation, the gut-derived metabolome, and urinary excretion (Suchy-Dicey et al., 2016; Bush et al., 2020). Protein binding is one reason secretion is clinically important. Only the unbound fraction of a solute is freely filtered at the glomerulus. Highly protein-bound uremic solutes are therefore inefficiently removed by filtration alone (Thompson and Joy, 2022). Tubular uptake can remove the free fraction from peritubular blood and may promote further dissociation from plasma proteins. In this way, secretion contributes to the elimination of solutes whose clearance is inadequately described by filtration alone (Suchy-Dicey et al., 2016; Alamilla-Sanchez et al., 2025). This is particularly relevant for gut-derived and protein-bound uremic solutes, such as indoxyl sulfate and p-cresyl sulfate, whose circulating levels may reflect renal clearance as well as nonrenal determinants, including diet, gut microbial metabolism, inflammatory milieu, and albumin binding (Viaene et al., 2013; Bush et al., 2020; Lowenstein and Nigam, 2021; Zaidan and Nazzal, 2022).

The same pathways also handle many drugs. Examples include loop and thiazide diuretics, beta-lactam antibiotics, some antiviral drugs, metformin, cimetidine, and other transporter substrates or inhibitors (Yin and Wang, 2016; Wang and Kestenbaum, 2018; Łapczuk-Romańska et al., 2023). This creates a potential for competition between retained uremic solutes and prescribed medications. It also means that drug exposure in CKD may reflect not only eGFR, but also residual tubular secretory capacity, transporter inhibition, and polypharmacy (Yin and Wang, 2016; Risso et al., 2019; Łapczuk-Romańska et al., 2023). In this sense, tubular secretion links solute retention with medication safety, making it clinically relevant even before it becomes a routine clinical test.

CKD can affect secretion via structural, metabolic, or circulating mechanisms. Nephron mass loss decreases the volume of secretory units. Tubular injury may dampen transporter levels and interfere with epithelial polarity (Wang and Kestenbaum, 2018; Risso et al., 2019). Solute delivery may be restricted by interstitial fibrosis and peritubular capillary rarefaction. Hypoxia and mitochondrial dysfunction can lead to decreased energy available for transport (Wang et al., 2022). Retention of uremic solutes (Prokopienko and Nolin, 2018; Spicher et al., 2025), polypharmacy (Yin and Wang, 2016; Łapczuk-Romańska et al., 2023), altered concentration of albumin, and altered binding of proteins (Celestin and Musteata, 2021) would modify the free fraction and transporter accessibility of secreted solutes and drugs. These structural, metabolic, and circulating changes provide several routes by which secretory clearance may diverge from filtration in CKD.

Together, these mechanisms support tubular secretion as a physiologically distinct component of kidney function. However, physiological distinction does not mean that secretion is independent of filtration in patients with CKD. Biological plausibility alone is not sufficient for clinical application. For secretion to become clinically useful in CKD, it must be measured reproducibly, interpreted consistently, and shown to add actionable information beyond eGFR and albuminuria.

## Assessing tubular secretion: analytical validity and current approaches

Analytical validity is the first translational requirement for tubular secretion testing (Hayes, 2015). For secretion, this means not only accurate quantification of selected solutes, but also clarity about what the measurement represents: renal clearance, relative excretion, circulating solute burden, or transporter-specific drug handling (Risso et al., 2019; Bullen et al., 2023). A clinically useful secretion measure should be reproducible, feasible across CKD stages, interpretable under standardized sampling conditions, and able to provide information beyond eGFR and albuminuria (Risso et al., 2019; Bullen et al., 2023).

Several approaches have been used to assess tubular secretion in humans. Timed renal clearance of endogenous secretory solutes provides a patient-level estimate of renal elimination. This approach requires paired plasma and urine measurements. When urine is collected over a defined period, clearance can be calculated from urine concentration, urine flow, and plasma concentration. However, renal clearance reflects net renal handling and does not isolate tubular secretion. Depending on the solute, measured clearance may include contributions from glomerular filtration and tubular reabsorption in addition to secretion (Thompson and Joy, 2022). Its interpretation as a secretory marker therefore depends on the extent to which the solute undergoes tubular secretion. Studies have used this approach to measure clearances of hippurate, cinnamoylglycine, indoxyl sulfate, p-cresyl sulfate, and other endogenous secretory solutes in CKD cohorts (Suchy-Dicey et al., 2016; Chen et al., 2020; Øvrehus et al., 2026). Accordingly, timed renal clearance provides a physiologically informative patient-level estimate of secretory handling, although it remains difficult to implement as a routine clinical test (Suchy-Dicey et al., 2016; Chen et al., 2020).

Targeted liquid chromatography–tandem mass spectrometry (LC-MS/MS) is the main analytical platform used in contemporary studies (Rodríguez-García et al., 2025; Øvrehus et al., 2026). It can measure multiple secretory solutes in plasma and urine. This makes multi-solute secretion panels possible. However, LC-MS/MS requires specialized equipment, standardized sample handling, calibration, quality-control procedures, and laboratory expertise (Suchy-Dicey et al., 2016; Chen et al., 2020; Rodríguez-García et al., 2025; Øvrehus et al., 2026). These requirements are feasible in research cohorts but remain difficult for routine clinical care. Thus, analytical performance depends not only on the assay platform but also on preanalytical handling, calibration standards, interlaboratory harmonization, and reproducibility across repeated measurements (Rivara et al., 2017; Rodríguez-García et al., 2025).

Solute selection is a major challenge because no single endogenous solute captures total proximal tubular secretion (Thompson and Joy, 2022; Bullen et al., 2023). Candidate solutes vary in transporter dependence, protein binding, metabolic origin, sensitivity to diet and gut microbial production, and the contribution of nonrenal clearance. Hippurate and cinnamoylglycine have been used as endogenous markers of secretion (Suchy-Dicey et al., 2016; Chen et al., 2020). Indoxyl sulfate and p-cresyl sulfate are clinically interesting because they are protein-bound and gut-derived. However, this also makes them harder to interpret. Their plasma levels may reflect impaired secretion, but also solute production, CKD severity, protein binding, microbiome activity, inflammation, and diet (Lowenstein and Nigam, 2021; Zaidan and Nazzal, 2022; Stepanova et al., 2024). Accordingly, individual solutes should not be treated as interchangeable measures of total tubular secretion. Instead, they are best considered as components of pathway-informed secretory profiles. The principal candidate biomarker classes and their translational characteristics are summarized in Box 1.

Box 1 **Candidate biomarkers of proximal tubular secretory function in CKD.**

Endogenous organic anions. Hippurate, cinnamoylglycine, kynurenic acid, xanthosine, isovalerylglycine, tiglylglycine, pyridoxic acid, and trimethyluric acid are the main candidate markers used in multi-solute secretion panels (Suchy-Dicey et al., 2016; Chen et al., 2020; Granda et al., 2023; Øvrehus et al., 2026). Their main value lies in estimating pathway-level secretory function rather than interpreting individual solutes in isolation.

Protein-bound uremic solutes. Indoxyl sulfate, p-cresyl sulfate, and indole-3-acetic acid provide clinically relevant information on the clearance of protein-bound and gut-derived compounds (Bush et al., 2020; Mair et al., 2021; Xie et al., 2025). Their interpretation is limited by protein binding, microbiome-dependent production, diet, and nonrenal metabolism.

Cationic transport markers. Creatinine reflects OCT2–MATE-mediated secretion in part, but its dominant dependence on glomerular filtration limits its specificity for tubular function (Yin and Wang, 2016; Łapczuk-Romańska et al., 2023). Other endogenous cationic markers remain insufficiently validated.

Drug probes. Metformin, cimetidine, and selected organic anion substrates can characterize transporter-specific function and drug–drug interactions (Chen et al., 2021a; Tan et al., 2022; Gessner et al., 2025), but their use requires controlled administration and pharmacokinetic sampling.

Most promising translational approach. A standardized multi-solute endogenous panel measured in paired plasma and urine samples is currently the most plausible strategy for clinical development. Such a panel will require assay harmonization, reproducibility testing, external validation, clinically meaningful thresholds, and demonstrated incremental value beyond eGFR and albuminuria.

These candidates should not be considered interchangeable because they capture different dimensions of tubular secretory function. Some primarily reflect pathway-level secretory clearance, whereas others are strongly influenced by solute production, protein binding, diet, microbiome composition, inflammation, or nonrenal metabolism.

Timed urine collection provides the most physiologically interpretable estimate of renal clearance of candidate secretory solutes (Suchy-Dicey et al., 2016; Chen et al., 2020). Its main limitation is feasibility. Timed collections are inconvenient and prone to incomplete collection. This can distort clearance estimates. Spot urine-to-plasma ratios are easier to obtain and more scalable. They may be useful in large cohorts, but they are more sensitive to urine concentration, hydration, sampling time, and short-term biological variation (Rivara et al., 2017; Ascher et al., 2022; Bullen et al., 2022). They should, therefore, be viewed as scalable surrogates of secretory handling rather than direct equivalents of timed clearance. Before clinical use, spot-based secretion scores will require standardized sampling conditions, normalization strategies, and repeatability testing (Garimella et al., 2017; Rivara et al., 2017).

Plasma concentrations of secretory solutes are easier to measure than clearance, but they should not be interpreted as direct measures of secretion. A high plasma level may reflect reduced renal clearance (Zaidan and Nazzal, 2022). It may also reflect increased production, altered diet, gut microbiome activity, inflammation, protein binding, nonrenal metabolism, or reduced removal in advanced kidney disease. This distinction is especially important for protein-bound and gut-derived uremic solutes (Rivara et al., 2017; Lowenstein and Nigam, 2021; Zaidan and Nazzal, 2022). Therefore, plasma-based measures are useful for characterizing uremic solute burden, but they cannot distinguish impaired tubular clearance from increased solute generation or altered protein binding without paired urine data.

Drug-probe approaches can test specific transporter pathways. In these studies, a probe substrate is administered and its pharmacokinetics are measured. This can provide information about organic anion, organic cation, or MATE-mediated transport (Gessner et al., 2025). However, these approaches are not practical for broad CKD assessment. They require drug administration, timed sampling, safety oversight, and pharmacokinetic modeling. They are useful for renal pharmacology and drug-interaction studies, but they are not ready as routine clinical tests (Gessner et al., 2025). Their main value is mechanistic: they can clarify transporter-specific function and drug-drug interaction liability, but they do not provide a simple secretion phenotype for routine CKD care.

Overall, current methods occupy different positions along the translational pathway. Timed collections estimate renal clearance of candidate secretory solutes, spot ratios provide scalable surrogate measures, plasma concentrations characterize circulating solute burden, and drug probes interrogate specific transporter mechanisms. Table 2 summarizes the main strengths, limitations, and potential applications of these approaches.

**TABLE 2:** Main approaches to assessing tubular secretion in human studies.

Approach	What it measures	Samples/platform	Strength	Main limitation	Current role in translation
Timed clearance of endogenous secretory solutes (Suchy-Dicey et al., 2016; Chen et al., 2020 ; Chen et al., 2021c)	Renal clearance of selected secreted solutes	Paired plasma and timed urine; usually LC-MS/MS	Quantitative patient-level estimate of renal clearance of candidate secretory solutes	Timed urine is inconvenient and prone to collection error. Requires specialized assays. May include contributions from filtration and, for some solutes, reabsorption	Physiology and cohort studies
Spot urine-to-plasma ratios or secretion scores (Garimella et al., 2017; Bhatraju et al., 2021; Bullen et al., 2022)	Relative excretion of selected secretory solutes	Paired spot urine and plasma; LC-MS/MS or metabolomics	More scalable than timed urine	Affected by urine concentration, hydration, sampling time, and short-term variability	Large observational cohorts and risk studies
Plasma concentration of secretory solutes (Rivara et al., 2017; Lowenstein and Nigam, 2021; Zaidan and Nazzal, 2022)	Circulating solute burden, not true clearance	Plasma or serum; LC-MS/MS, HPLC, or metabolomics	Simple sampling. Useful for uremic burden	Affected by production, diet, microbiome, protein binding, inflammation, nonrenal metabolism, and clearance	Studies of uremic solute burden and toxicity
Free and total protein-bound solute measurement (Suchy-Dicey et al., 2016; Mair et al., 2021; Rodríguez-García et al., 2025)	Total and unbound fractions of protein-bound uremic solutes	Plasma or serum; ultrafiltration plus LC-MS/MS or HPLC.	Shows biologically available free fraction	Technically sensitive. Sample handling can affect results	Protein-bound uremic toxin and dialysis studies
Drug-probe pharmacokinetic and transporter-probe studies (Chen et al., 2021a; Tan et al., 2022; Gessner et al., 2025)	Transporter-dependent drug handling	Probe drug administration with timed plasma and/or urine sampling	Can test specific transporter pathways and drug-drug interactions	Requires drug administration, safety oversight, timed sampling, and modeling. Not validated for routine dose adjustment	Renal pharmacology and transporter interaction studies
*In vitro* transporter assays (Yin and Wang, 2016; Łapczuk-Romańska et al., 2023; Thakur et al., 2025)	Transporter specificity, substrate affinity, or inhibition	Transporter-expressing cells, vesicles, or membrane systems	Defines mechanism and supports interpretation of transporter pathways	Does not measure secretion in a patient. Translation to CKD is indirect	Mechanistic support, drug development, and DDI prediction
Integrated endogenous solute panel (Chen et al., 2020; Granda et al., 2023; Øvrehus et al., 2026)	Composite estimate of secretion across several solutes	Plasma and urine panel; LC-MS/MS or metabolomics	May capture secretion better than a single marker	Needs standard solute selection, assay harmonization, reproducibility testing, thresholds, and outcome validation	Translational biomarker development

Abbreviations: CKD, chronic kidney disease; DDI, drug-drug interaction; HPLC, high-performance liquid chromatography; LC-MS/MS, liquid chromatography–tandem mass spectrometry.

Taken together, these methods capture complementary but noninterchangeable dimensions of tubular secretion, ranging from patient-level clearance and circulating solute burden to transporter-specific mechanisms. The most promising translational approach is likely a standardized endogenous solute panel with defined sampling conditions, assay harmonization, reproducibility metrics, reference intervals or clinically meaningful thresholds, and demonstrated incremental value beyond eGFR and albuminuria (Chen et al., 2020; Ascher et al., 2022; Øvrehus et al., 2026).

Thus, the central measurement problem is not whether tubular secretion can be quantified, but whether it can be quantified in a way that is reproducible, interpretable, and clinically comparable across studies and CKD populations. This analytical foundation is necessary before secretion measures can be evaluated for clinical validity.

## Clinical validity: associations with kidney, cardiovascular, and safety outcomes

After secretion can be measured, the next question is whether it is clinically meaningful. Clinical validity refers to associations between secretory measures and relevant outcomes, whereas clinical utility requires evidence that testing changes management or improves outcomes. Current evidence supports prognostic relevance, particularly for kidney outcomes, but remains insufficient for routine clinical decision-making.

The strongest evidence comes from studies of kidney outcomes. In the Chronic Renal Insufficiency Cohort (CRIC) study, lower kidney clearances of endogenous secretory solutes remained associated with CKD progression and all-cause mortality after adjustment for eGFR and albuminuria (Chen et al., 2020). This finding suggests that secretory measures may capture risk information not fully represented by eGFR and albuminuria. However, persistence of an association after statistical adjustment does not establish that tubular secretion is physiologically independent of glomerular function or that it provides incremental predictive value. Moreover, the associations were not uniform across all solutes, and the study was observational. Therefore, these data support clinical validity, but they do not yet establish clinical utility. Importantly, the CRIC analysis used plasma and 24-h urine measurements, making it one of the more physiologically informative human studies of renal clearance of secretory solutes.

Other studies also suggest that secretory measures may identify the risk of kidney function decline. In the Systolic Blood Pressure Intervention Trial (SPRINT), lower estimated tubular secretion, assessed using spot urine-to-plasma ratios of endogenous markers, was associated with faster eGFR decline among participants with CKD, independent of baseline eGFR and albuminuria (Ascher et al., 2022). In the Jackson Heart Study, lower tubular secretory clearance was associated with a greater risk of subsequent eGFR decline in a community-based cohort (Granda et al., 2022). These findings are important because they were observed using different study designs and populations. They suggest that impaired secretion may reflect kidney vulnerability not fully represented by eGFR at a single time point. More recent prospective data also support the clinical relevance of protein-bound secretory solute clearance. In non-dialysis CKD, lower 24-h kidney clearances of indoxyl sulfate and indole-3-acetic acid were independently associated with renal adverse outcomes and hospitalization (Xie et al., 2025). Although this study was smaller and solute-specific, it strengthens the kidney-outcome signal by using clearance-based assessment rather than plasma concentration alone. However, all these studies also illustrate a key limitation of the field: secretion has been estimated using different solutes, collection methods, and summary measures, making direct comparison across cohorts difficult.

The relationship between secretory clearance and cardiovascular outcomes is less clear. In a CRIC cardiovascular analysis, lower clearances of several secretory solutes and a lower summary secretion score were associated with incident heart failure and myocardial infarction in models before full adjustment for eGFR. After adjustment for eGFR, the associations with heart failure, myocardial infarction, and stroke were attenuated and were not clinically or statistically robust (Chen et al., 2021b). A plausible explanation is that reduced tubular secretion partly reflects the overall severity of CKD. Loss of functioning nephron mass and tubulointerstitial injury can affect both filtration and tubular secretory capacity (Wang and Kestenbaum, 2018; Mair et al., 2021). In advanced CKD, secretory clearances of several organic solutes are markedly reduced and may decline more than eGFR (Mair et al., 2021). Lower secretory clearance may therefore identify patients with more severe kidney dysfunction without representing a separate cardiovascular pathway. The attenuation after adjustment for eGFR supports a cautious interpretation of any additional cardiovascular information provided by secretory measures. Thus, cardiovascular findings should be interpreted as hypothesis-generating rather than sufficient evidence for secretion-based cardiovascular risk stratification.

Studies of adverse events and medication-related vulnerability may be especially relevant to clinical translation. In SPRINT participants with CKD, a lower tubular secretion score was associated with a higher risk of a composite adverse event outcome, including acute kidney injury, electrolyte abnormalities, hypotension, syncope, bradycardia, injurious falls, hyperkalemia, and hypokalemia (Bullen et al., 2022). The association remained after adjustment for eGFR and albuminuria. In analyses of individual outcomes, the associations appeared strongest for acute kidney injury, serious electrolyte abnormalities, and ambulatory hyperkalemia (Bullen et al., 2022). These findings are clinically plausible because tubular secretion is closely linked to drug handling, solute clearance, and tubular homeostatic function. They also identify medication safety and treatment tolerance as potentially actionable use cases for future secretion-guided studies. However, the outcome was composite, the study was observational, and it did not test whether knowledge of secretion status would have changed management or prevented adverse events.

Advanced CKD studies provide complementary mechanistic evidence. In patients with advanced CKD, secretory clearances for many organic solutes may be reduced more than GFR (Mair et al., 2021). This suggests that secretion can be impaired out of proportion to filtration. Such divergence may contribute to the retention of protein-bound and actively secreted solutes as patients approach kidney failure. However, these studies are generally smaller and are better viewed as mechanistic support rather than direct evidence for clinical implementation. Their value is that they strengthen biological plausibility by showing that secretory failure may become disproportionate to filtration loss in advanced disease.

Not all outcome studies show consistent associations. For example, in the REGARDS study, baseline estimated tubular secretion was not associated with subsequent sepsis-associated acute kidney injury (Bullen et al., 2025). This negative finding is important because it shows that secretion measures may not predict all kidney-related outcomes equally and that associations may depend on outcome type, population, timing of measurement, and the secretion phenotype used. Future studies should therefore define the clinical context in which secretion is expected to add value rather than assuming that lower secretion is a universal risk marker.

Table 3 summarizes the main human studies linking tubular secretion measures with clinical outcomes.

**TABLE 3:** Human studies linking tubular secretion measures with clinical outcomes.

Study	Population	Secretory measure	Measurement approach	Outcome domain	Main finding
Suchy-Dicey et al. (2016)	298 patients with kidney disease	Hippurate, cinnamoylglycine, p-cresyl sulfate, indoxyl sulfate	Serum plus timed urine samples; LC-MS/MS; clearance calculation	Mortality, CKD progression to dialysis	Low hippurate or p-cresyl sulfate clearance was associated with higher risk of death, independent of eGFR; low cinnamoylglycine clearance showed a possible association with dialysis risk
Chen et al. (2020)/CRIC, 2020	3,416 adults with CKD in CRIC	LC-MS/MS panel of endogenous secretory solutes, including kynurenic acid, pyridoxic acid, indoxyl sulfate, xanthosine, isovalerylglycine, tiglylglycine, hippurate, and trimethyluric acid	Plasma plus 24-h urine; kidney clearance calculated for each solute	CKD progression, mortality	Lower clearances were associated with CKD progression and all-cause mortality independent of eGFR and albuminuria
Chen et al. (2021a)/CRIC metabolic analysis Chen et al. (2021c)	Adults with CKD in CRIC	Endogenous secretory solutes, mainly organic anions	eGFR compared with tubular solute clearances	Metabolic complications	Secretory clearances were correlated with eGFR and associated with selected metabolic complications
Chen et al. (2021a)/CRIC cardiovascular analysis Chen et al. (2021b)	3,407 participants with CKD from CRIC	Eight endogenous secretory solutes measured by LC-MS/MS	Plasma and 24-h urine; kidney clearances estimated from LC-MS/MS data	Incident heart failure, myocardial infarction, stroke	Lower 24-h kidney clearances of secretory solutes were associated with incident heart failure and myocardial infarction, but not stroke; these associations were attenuated after adjustment for eGFR
Bhatraju et al. (2021)	170 critically ill patients and 70 healthy controls	7 endogenous secretory solutes, including isovalerylglycine and tiglylglycine	Paired spot urine and plasma; urine-to-plasma ratios and composite secretion score	28-day major adverse kidney events, mortality-related outcomes	Lower urine-to-plasma ratios and lower secretion score were associated with worse short-term outcomes
Ascher et al. (2022)/SPRINT	2,089 participants with CKD	Estimated secretion score based on endogenous secretory solutes	Paired spot urine and plasma; urine-to-plasma ratios used to derive estimated tubular secretion	eGFR decline, CKD progression, CVD, mortality	Lower secretion was associated with faster eGFR decline, but not independently with CKD progression, CVD, or mortality
Bullen et al. (2022)/SPRINT	SPRINT participants with CKD	Endogenous secretion markers summarized as a secretion score	Paired spot urine and plasma; urine-to-plasma ratios	Adverse events, including AKI and electrolyte abnormalities	Worse secretion was associated with higher adverse-event risk independent of eGFR and albuminuria
Granda et al. (2022)/Jackson Heart Study	254 participants in 127 matched pairs of African American adults; matched on creatinine-eGFR, age, diabetes, and sex	5 endogenously produced secretory solutes: isovalerylglycine, kynurenic acid, xanthosine, hippurate, and one additional endogenous secretory solute assessed in the study	Baseline plasma and 24-h urine concentrations measured by LC-MS/MS	Longitudinal eGFR decline	Lower kidney clearance of isovalerylglycine, kynurenic acid, and xanthosine was associated with higher odds of eGFR decline; kynurenic acid had the strongest association, with each 50% lower clearance linked to 2.20-fold higher odds of eGFR decline
Bullen et al. (2025)/REGARDS	352 participants from the REGARDS study	Estimated kidney tubular secretion score	Baseline ambulatory paired blood/urine secretion markers; summary secretion score	Sepsis-associated AKI	Baseline secretion score was not associated with sepsis-associated-AKI.
Xie et al. (2025)	186 non-dialysis patients with CKD followed prospectively	Protein-bound uremic toxins: indoxyl sulfate, p-cresyl sulfate, and indole-3-acetic acid	Serum and 24-h urine measurements; 24-h kidney clearances and fractional clearances	Renal adverse outcomes and hospitalization	Lower 24-h kidney clearances of indoxyl sulfate and indole-3-acetic acid were independently associated with higher risk of renal adverse outcomes and hospitalization

Abbreviations: AKI, acute kidney injury; CKD, chronic kidney disease; CRIC, chronic renal insufficiency cohort; CVD, cardiovascular disease; eGFR, estimated glomerular filtration rate; LC-MS/MS, liquid chromatography–tandem mass spectrometry; REGARDS, reasons for geographic and racial differences in stroke; SPRINT, systolic blood pressure intervention trial.

Overall, tubular secretion is supported as a clinically relevant research measure, with the most consistent associations observed for kidney function decline and CKD progression. Evidence for mortality requires confirmation; cardiovascular associations are attenuated after adjustment for eGFR, and adverse-event findings remain promising but observational. Differences in solute panels, sampling strategies, and outcome definitions further limit comparisons across studies. Thus, current evidence supports clinical validity but not clinical utility. This translational gap extends beyond tubular secretion and is shared by biomarkers of tubular injury, inflammation, fibrosis, endothelial dysfunction, oxidative stress, and uremic solute burden. Across these domains, biological relevance and associations with CKD progression or complications are insufficient for clinical implementation unless a biomarker provides incremental information beyond established kidney measures, informs a predefined clinical decision, or improves patient outcomes (Gutiérrez et al., 2022; Demikhova et al., 2025; Dopierała et al., 2025). The next step is therefore to determine whether secretory measures improve prediction, identify actionable patient subgroups, or guide management in ways that benefit patients.

## From clinical validity to clinical utility: a translational roadmap

**FIGURE 2:**
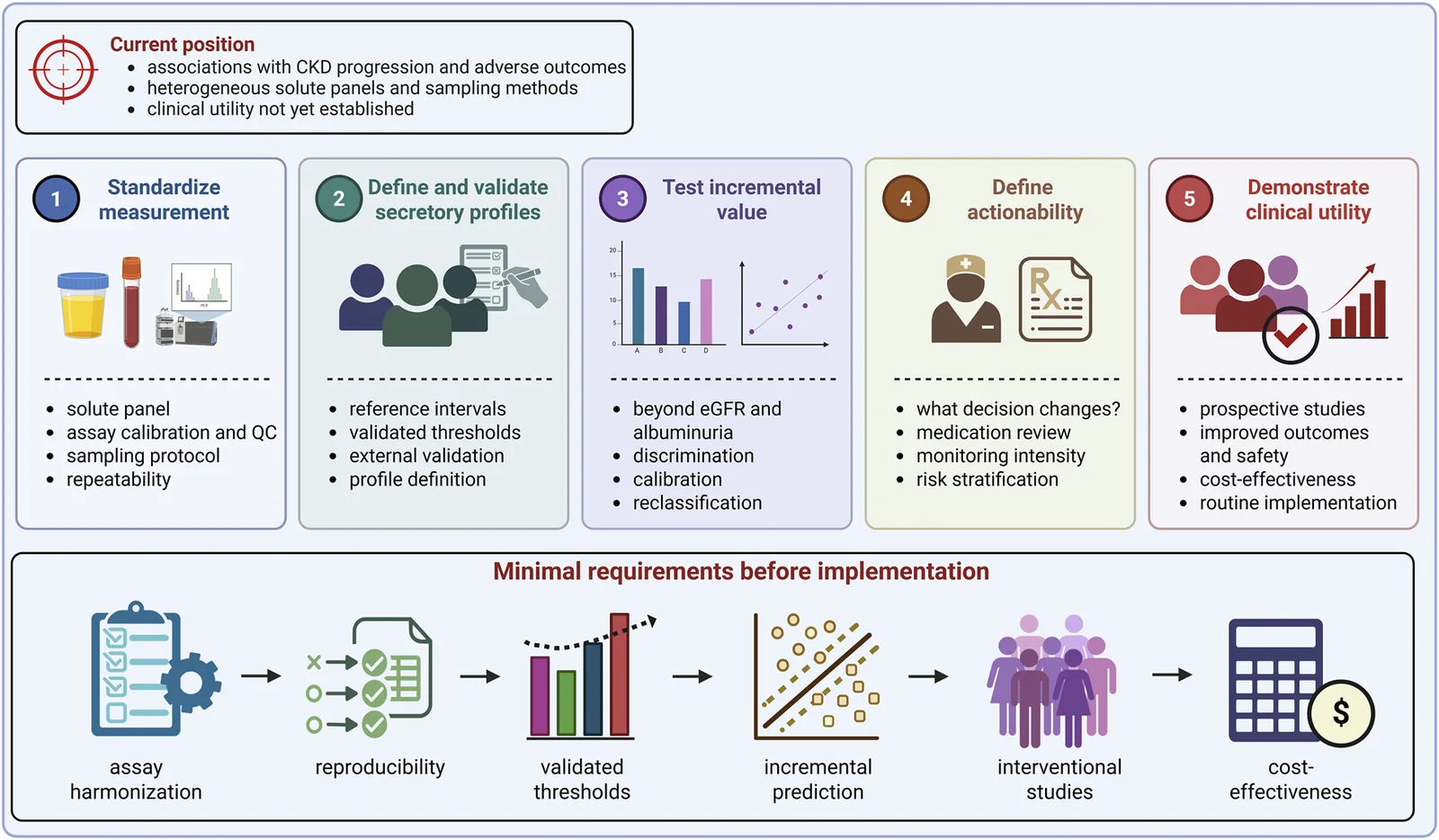
Translational roadmap for defining clinically useful secretory profiles in CKD. The figure summarizes the steps required to move from research measurement to clinical implementation: standardization of secretory assays, definition and validation of secretory profiles, testing of incremental value beyond eGFR and albuminuria, identification of actionable clinical decisions, and prospective demonstration of clinical utility. This framework treats secretory profiling as a candidate clinical tool whose value must be demonstrated before implementation, not as an established CKD classification system. Created in BioRender. Stepanova, N. (2026) https://BioRender.com/3fng4ff License: BioRender Academic Publication License, Agreement No. DU2A5UKS9R Source: BioRender.

The next translational step is to determine whether measuring tubular secretion adds actionable information beyond standard CKD assessment. Current barriers include heterogeneous solute panels, nonstandardized sampling, inconsistent summary scores, undefined thresholds, and uncertain incremental value beyond established CKD predictors. Figure 2 summarizes the proposed pathway from standardized measurement to prospective demonstration of clinical utility.

The first requirement is assay standardization. A clinically useful secretion test will require a reproducible solute panel that is small enough for clinical use but broad enough to reflect major proximal tubular transport pathways. Because no single endogenous solute captures total tubular secretion, multi-solute panels are likely to be more informative than single-marker approaches (Wang and Kestenbaum, 2018; Granda et al., 2023). Candidate solutes should be selected with attention to transporter dependence, protein binding, diet sensitivity, gut microbial production, nonrenal metabolism, and assay reproducibility (Wang and Kestenbaum, 2018; Granda et al., 2023; Spicher et al., 2025).

The second requirement is sampling harmonization. Timed urine collections provide the most physiologically interpretable estimates of secretory clearance, but they are difficult to implement in routine care (Suchy-Dicey et al., 2016; Chen et al., 2020). Spot urine-to-plasma ratios are more scalable, but require standardized collection conditions, normalization strategies, and repeatability testing because they are affected by hydration, urine concentration, sampling time, and short-term biological variation (Rivara et al., 2017; Ascher et al., 2022; Alamilla-Sanchez et al., 2025). Plasma concentrations are simpler to measure, but primarily reflect circulating solute burden rather than secretory clearance (Wang and Kestenbaum, 2018; Spicher et al., 2025). Therefore, future studies should define which sampling strategy is appropriate for each intended use case.

The third requirement is validation of secretory profiles as clinically meaningful phenotypes. Secretory phenotypes should be tested across CKD stages, etiologies, comorbidity profiles, medication exposures, and populations. CKD etiology may affect tubular secretion. In an outpatient CKD cohort of 223 patients, several secretory solute clearances differed by CKD etiology, with higher clearances in glomerular disease and diabetic kidney disease than in vascular kidney disease (Wang et al., 2020b). Lower fractional excretion of several secretory solutes has also been reported in autosomal dominant polycystic kidney disease, including patients with preserved eGFR (Wang et al., 2020a). Age-related differences are less clear. In 636 healthy adults from the HUNT3 study, reference intervals were similar across age and sex (Øvrehus et al., 2026). Analytical performance, reference intervals, prognostic value, and clinically actionable thresholds should therefore be validated across CKD etiologies and relevant populations before routine implementation.

Genetic factors may also contribute to variation in tubular transport. Inherited tubulopathies show that defects in specific renal transport proteins can produce distinct solute-handling phenotypes (Downie et al., 2021). Genetic variation may also affect secretory transporters. Rare variants in OAT1/SLC22A6 and OAT3/SLC22A8 have been identified in patients with hyperuricemia and gout, and functional testing showed reduced urate transport for one OAT3 variant (Vávra et al., 2022). However, such effects are transporter- and substrate-specific and do not represent global proximal tubular secretory function. At present, evidence is insufficient to select secretory biomarker panels based on family history or genotype. Genetic factors may instead be considered potential modifiers in future validation studies.

A clinically meaningful secretion phenotype should identify tubular vulnerability that is not apparent from eGFR and albuminuria alone (Wang and Kestenbaum, 2018; Chen et al., 2020; Bullen et al., 2022). This phenotype may be most relevant when uremic solute burden, medication intolerance, or adverse-event risk appears disproportionate to standard CKD markers (Mair et al., 2021; Ascher et al., 2022; Spicher et al., 2025).

The fourth requirement is incremental prediction. Secretory measures should improve risk assessment beyond established predictors, including eGFR, albuminuria, age, diabetes, blood pressure, cardiovascular disease, and medication burden (KDIGO CKD Work Group, 2024). Improvement should be tested using discrimination, calibration, reclassification, and decision-curve analyses (Vickers and Holland, 2021; Binuya et al., 2022). Statistical association alone is not sufficient. A secretion score should identify patients for whom clinical decisions would change. Artificial intelligence (AI) and machine-learning methods may help integrate multi-solute secretion profiles with clinical variables and identify patterns that are difficult to capture with conventional models. However, AI-based tools would require transparent reporting, external evaluation across populations, and prospective evidence of clinical utility before they could support routine CKD care (Collins et al., 2024; Lekadir et al., 2025).

The fifth requirement is clinical actionability. A secretion result should be linked to a decision that could plausibly change care, such as medication review, dose adjustment for drugs with substantial transporter-mediated renal elimination, avoidance of transporter-mediated drug interactions, closer monitoring for adverse events, intensified follow-up, or enrichment of clinical trials with patients who have tubular vulnerability (Hayes, 2015; Mihaila et al., 2020; Vickers and Holland, 2021; Łapczuk-Romańska et al., 2023; Spicher et al., 2025). Without such a decision point, even a reproducible and prognostic secretion measure would remain a research biomarker rather than a clinical tool.

Medication safety is the most plausible first clinical use case. Many drugs used in CKD depend on tubular transport, including diuretics, antibiotics, antiviral drugs, metformin, and other transporter substrates or inhibitors (Wang and Kestenbaum, 2018; Łapczuk-Romańska et al., 2023). Clinical pharmacokinetic studies provide a more direct link between tubular secretion and drug elimination. In stable outpatients with a wide range of kidney function, endogenous secretion measures predicted kidney clearance of furosemide and penciclovir. Measured GFR showed similar predictive accuracy, and combining GFR with secretory measures produced only modest improvement (Chen et al., 2021a). Pharmacokinetic analysis of 33 OAT1/3 substrates also suggested that active secretion may decline more than GFR in severe CKD, which may affect prediction of renal drug clearance and dose requirements (Tan et al., 2022). In patients with impaired secretion, retained uremic solutes and polypharmacy may compete for shared transport pathways, potentially altering drug and uremic solute handling (Mihaila et al., 2020; Spicher et al., 2025). However, evidence that uremic solute–drug competition changes drug exposure or clinical outcomes in patients with CKD remains limited. A prospective secretion-guided strategy could test whether identifying impaired secretory capacity improves medication safety during treatment with transporter-dependent or interacting drugs.

Risk stratification is another potential application, but it requires evidence beyond association. Secretory profiling may help identify patients at higher risk of CKD progression or adverse events, support individualized follow-up, or enrich trials with patients who have tubular vulnerability (Chen et al., 2020; Ascher et al., 2022; Bullen et al., 2022; Granda et al., 2022). However, clinical implementation should not occur before the benefit is proven. A biomarker that improves prediction is not automatically useful in practice (Vickers and Holland, 2021; Binuya et al., 2022).

Prospective utility testing is the final requirement. Before routine implementation, secretion-guided strategies should be tested against usual care for their effects on patient outcomes, medication safety, risk stratification, and cost-effectiveness (Pletcher and Pignone, 2011; Hayes, 2015). Each study should define the intended clinical context, the secretion measure being tested, the decision triggered by the result, and the outcome expected to improve (Hayes, 2015; Vickers and Holland, 2021). Only with this evidence can tubular secretion move from clinical validity to clinical utility in CKD care.

## Conclusion

Over the last decade, tubular secretion has moved from a mainly physiological concept to a measurable functional domain of kidney health in CKD. Human cohort studies have shown that secretory clearance can be quantified and has been associated with CKD progression, mortality, and selected adverse events. These findings support the clinical validity of tubular secretion as a research measure. However, they do not yet establish clinical utility.

The next stage of research should be focused less on proving that secretion can be measured and more on defining when measurement matters. The priority is to define a reproducible secretion panel, test its added value, and determine whether secretion-guided decisions improve outcomes. Until such evidence is available, tubular secretion should be considered a promising translational biomarker rather than a routine clinical test. Its future role will depend on whether it can move from measurable biology to actionable clinical use. If successful, it may help shift CKD assessment from a filtration-centered model toward a more complete functional model of kidney health.
